# The Analyzation of Change in Documentation due to the Introduction of Electronic Patient Records in Hospitals—A Systematic Review

**DOI:** 10.1007/s10916-022-01840-0

**Published:** 2022-07-04

**Authors:** Florian Wurster, Garret Fütterer, Marina Beckmann, Kerstin Dittmer, Julia Jaschke, Juliane Köberlein-Neu, Mi-Ran Okumu, Carsten Rusniok, Holger Pfaff, Ute Karbach

**Affiliations:** 1 Faculty of Human Sciences & Faculty of Medicine and University Hospital Cologne, Institute of Medical Sociology, Health Services Research, and Rehabilitation Science, University of Cologne, Cologne, Germany; 2 Center for Health Economics and Health Services Research, University of Wuppertal, Wuppertal, Germany

**Keywords:** Digital transformation, Electronic patient record, Documentation, Hospital, Systematic review

## Abstract

The major impact on healthcare through the ongoing digital transformation and new technologies results in opportunities for improving quality of care. Electronic patient records (EPR) are a substantial part in this transformation, even though their influence on documentation remains often unclear. This review aims to answer the question of which effect the introduction of the EPR has on the documentation proper in hospitals. To do this, studies are reviewed that analyze the documentation itself, rather than merely conducting interviews or surveys about it. Several databases were searched in this systematic review (PubMed including PubMed, PubMed Central and Medline; PDQ Evidence; Web of Science Core Collection; CINHAL). To be included, studies needed to analyze written documentation and empirical data, be in either German or English language, published between 2010 and 2020, conducted in a hospital setting, focused on transition from paper-based to electronic patient records, and peer reviewed. Quantitative, qualitative and mixed methods studies were included. Studies were independently screened for inclusion by two researchers in three stages (title, abstract, full text) and, in case of disagreement, discussed with a third person from the research team until consensus was reached. The main outcome assessed was whether the studies indicated a negative or positive effect on documentation (e.g. changing the completeness of documentation) by introducing an EPR. Mixed Methods Appraisal Tool was used to assess the individual risk of bias in the included studies. Overall, 264 studies were found. Of these, 17 met the inclusion criteria and were included in this review. Of all included studies, 11 of 17 proved a positive effect of the introduction of the EPR on documentation such as an improved completeness or guideline adherence of the documentation. Six of 17 showed a mixed effect with positive and negative or no changes. No study showed an exclusively negative effect. Most studies found a positive effect of EPR introduction on documentation. However, it is difficult to draw specific conclusions about how the EPR affects or does not affect documentation since the included studies examined a variety of outcomes. As a result, various scenarios are conceivable with higher or reduced burden for practitioners. Additionally, the impact on treatment remains unclear.

**Supplementary Information:**

The online version contains supplementary material available at 10.1007/s10916-022-01840-0.

## Introduction

The ongoing digital transformation is having a major impact on healthcare. New technologies offer great opportunities to improve the quality of care. Electronic patient records (EPR) are key components for the digital transformation in hospitals and determine several clinical elements like communication and collaboration, information availability and workflows [1]. There is evidence for improved coordination of care and therefore higher quality of care which is contrasted with certain aspects of the EPR that may lead to higher staff burden [2, 3]. Despite the impact that the implementation of an EPR has been shown to have, clinical documentation itself is often not included in investigations. However, certain analyzable aspects of documentation like completeness, accuracy or legibility have been proposed since the emergence of EPRs [4]. Ignoring possible changes in documentation due to the introduction of an EPR seems doubtful, since inadequate documentation of clinically relevant aspects could result in patients not receiving the treatments they need [5, 6]. This review follows the research question of which effect the introduction of the EPR has on the actual clinical documentation in hospitals and summarizes evidence from the comparison of paper-based and electronic patient records.

## Methods

To shed light on the research question, a systematic review was conducted and is reported based on the most recent version of the “Preferred Reporting Items for Systematic Reviews and Meta-Analyses” (PRISMA) guidelines described by Page et al. whenever it is applicable [7]. See Online Resource 1 for a detailed list where to find which items.

### Search Strategy & Selection Criteria

Following a sensitive search strategy to identify all suitable studies, several electronic bibliographic databases were searched including PubMed (incl. PubMed, PubMed Central, MEDLINE), Web of Science Core Collection, CINAHL, and PDQ Evidence. The components for the database search were “implementation”, “electronic patient record”, “paper-based”, “documentation”, and “hospital”. For all databases, filters were used to limit the results to English and German language and the period of publication from 2010–2020. The period of publication was limited as previous, unsystematic research showed that some studies from before 2010 examined technologies that are no longer in use today due to the rapid progress of digital systems. See Online Resource 2 for the detailed search strategies whose construction was not accompanied by a librarian. Synonyms, Boolean Operators, number of results, date, and filters or special features like truncations for all databases can be found there.

Screening of results was conducted in three steps by three researchers (FW, GF, UK) with inclusion or exclusion of studies following the criteria in Table 1. At this point, it should be emphasized that the focus of this systematic review is on the documentation itself and not on the results of interviews or surveys about it. According to point 5 in Table 1, only studies that analyzed actual patient records were included. In the first step, all titles were screened independently by FW and GF. Thereupon, abstracts were screened independently by FW and GF resulting in screening of the remaining full texts by FW and GF. Discrepancies in the first two steps meant including the studies in the next step until enduring discrepancies were discussed in the last step together with UK and consensus was reached. Screening was conducted in all steps following a questionnaire (see Online Resource 3) that covered all inclusion criteria.

**Table 1:** Inclusion and exclusion criteria

**Inclusion Criteria**	**Exclusion Criteria**
English or German language	A language other than German or English
Publication period from 2010—2020	Published before 2010
Hospital setting	Ambulatory setting, outpatient clinics, nursing homes, rehabilitation centers, intersectoral care
Focus on the transition from paper-based to electronic patient records	Exclusive consideration of paper or exclusive consideration of electronic documentation
Document analysis of written documentation	Interviews or surveys about documentation
Analysis of data	Secondary literature like reviews, comments, essays
Peer reviewed publication	Thesis, newspaper articles, symposia

### Data Items & Collection Process

The extracted data included authors, year, country, setting, study design, number of analyzed records, outcomes, results, and, if applicable, a use case. The outcomes were classified into the framework given by Nonnemacher, Nasseh, and Stausberg regarding their dimension of quality, meaning that e.g. the analysis of the outcome usage of standardized nursing language could be assigned to the dimension of structural quality [8]. For quantitative studies, statistical numbers like confidence intervals, p-values, or other relevant effect measurements were also extracted. See Table 2 for study characteristics, Table 3 for outcomes and results and Table 4 for study designs which allows a clear overview of the results of the individual studies, potential missing data and heterogeneity of the included studies. Included publications were stored in a Citavi library and extracted data was summarized in Microsoft Excel.

**Table 2:** Study characteristics

**Authors**	**Country**	**Setting**	**Use Case**	**N**
Al Muallem et al. [21]	Saudi Arabia	Radiology department at a military hospital	Medical imaging referral forms	456 documents (228 paper records vs. 228 electronic records)
Barritt et al. [13]	United Kingdom	Orthopedic surgical ward	Operation reports for unilateral hip hemiarthroplasty	80 documents (50 paper records vs. 30 electronic records)
Bell et al. [18]	USA	Emergency department in one 793 bed hospital	Discharge instructions	300 documents (150 paper records vs. 150 electronic records)
Boo et al. [16]	South-Korea	700 bed academic teaching hospital	Chief complaint and present illness	2,281 documents (1,159 paper records vs. 1,122 electronic records)
Bruylands et al. [10]	Switzer-land	Midsized general hospital	Nursing diagnoses	108 documents (36 paper records vs. 36 paper records vs. 36 electronic records)
Choi et al. [22]	USA	705 bed teaching hospital	Preoperative screening, preanesthesia evaluation, perioperative care, postoperative phases	4,981 documents (3,997 paper records vs. 984 electronic records)
Coffey et al. [23]	USA	Pediatric level I trauma center	Trauma resuscitation	400 documents (200 paper records vs. 200 electronic records)
Hampe et al. [14]	USA	Burn unit at a tertiary hospital	Lund Browder documentation for burn wound classification	not specified
Jamieson et al. [11]	Canada	Internal medicine unit at a large urban academic teaching hospital	Admission notes	42 documents (21 paper records vs. 21 electronic records)
Jang et al. [15]	South-Korea	1200 bed hospital	Anesthesia records	250 documents (100 paper records vs. 150 electronic records)
Liu and Edye [12]	Australia	Large sub-tertiary hospital	Appendicectomies	318 documents (98 paper records vs. 107 electronic records t1 vs. 113 electronic records t2)
Lucas et al. [24]	Germany	Level 1 trauma center at the emergency department at an academic teaching hospital	Traumatological patients	10,891 documents (3,199 paper records vs. 2,910 electronic records t1 vs. 4,782 electronic records t2)
McCamley et al. [25]	Australia	900-bed tertiary academic teaching hospital	Nutrition data & dietetic chart	312 documents (183 paper records vs. 129 electronic records) & 8 paper audits incl. 3,834 records vs. 5 electronic audits incl. 2,958 records
Montagna et al. [19]	Italy	Trauma center	Trauma resuscitation	40 documents (20 paper records vs. 20 electronic records)
Thoroddsen et al. [26]	Iceland	800 bed university hospital with 50 wards (41 sampled)	Nursing care plans	580 documents (299 paper records vs. 281 records (195 electronic & 86 paper))
Yadav et al. [17]	USA	not specified	Physical examination in initial progress notes of 5 ICD-9 diagnoses	500 documents (250 paper records vs. 250 electronic records)
Zargaran et al. [20]	South Africa	Academic tertiary referral trauma hospital	Admission notes, operative notes, and discharge summaries of patients requiring full trauma team activation	20,848 documents (9,236 paper records vs. 11,612 electronic records)

**Table 3:** Key results

**Authors**	**Outcome**	**Key Result**	**Effect**
Al Muallem et al. [21]	Completeness, Legibility	Electronic documentation significantly improved completeness (*p* < 0.001) and legibility (*p* < 0.001)	+
Barritt et al. [13]	Guideline adherence	Electronic documentation significantly improved guideline adherence (*p* < 0.01)	+
Bell et al. [18]	Guideline adherence	Electronic documentation significantly improved guideline adherence (*p* < 0.05)	+
Boo et al. [16]	Volume of documentation	Electronic documentation did not change volume of documentation in chief complaint and present illness measured by normalized bytes. When measured by number of words, volume of documentation in chief complaint did not change, while volume of documentation in present illness decreased (*p* < 0.03)	~
Bruylands et al. [10]	Standardized Nursing Language	Electronic documentation showed higher rates of standardized nursing language	+
Choi et al. [22]	Guideline adherence	Electronic documentation significantly improved guideline adherence (*p* < 0.001)	+
Coffey et al. [23]	Completeness	Electronic documentation significantly improved completeness in 5 out of 11 elements (*p* < 0.001 & *p* < 0.05) but significantly worsened completeness in 1 out of 11 elements (*p* < 0.001)	~
Hampe et al. [14]	Guideline adherence	Electronic documentation improved guideline adherence	+
Jamieson et al. [11]	Quality of documentation, Volume of documentation	Electronic documentation significantly improved quality of documentation (*p* < 0.0001) but free-text subsections were significantly longer (*p* < 0.0001)	~
Jang et al. [15]	Completeness	Electronic documentation significantly improved overall completeness (*p* < 0.01) but only in the automatically, not in the manually documented items	~
Liu and Edye [12]	Quality of documentation	Electronic documentation significantly improved quality of documentation (*p* = 0.001)	+
Lucas et al. [24]	Structured documentation	Electronic documentation significantly improved usage of structured documentation in 18 of 20 information fields (*p* < 0.05)	+
McCamley et al. [25]	Completeness, Legibility	Electronic documentation significantly improved legibility (*p* < 0.001) and completeness (*p* < 0.01)	+
Montagna et al. [19]	Volume of documentation, Accuracy	Electronic documentation improved accuracy but was longer. Documentation style changed from a narrative first-person style to a list of events, including time and place	~
Thoroddsen et al. [26]	Completeness, Standardized nursing language	Electronic documentation showed significantly higher rates of standardized nursing language (*p* < 0.001) and improved completeness	+
Yadav et al. [17]	Completeness, Accuracy, Inaccuracy, Volume of Documentation	Electronic documentation showed a significantly higher rate of inaccuracy (*p* < 0.001) with higher rate of completeness (*p* < 0.001). Electronic documentation was significantly longer (*p* < 0.001)	~
Zargaran et al. [20]	Completeness	Electronic documentation significantly improved completion in admission notes, operative notes, and discharge summaries (for all comparisons, *p* < 0.001)	+

**Table 4:** MMAT ratings

**Authors**	**Qualitative Study**	**Mixed Methods Study**	**Quanti-tative Descriptive Study**	**Non-Rando-mized Study**	**Rando-mized Controlled Study**	**MMAT-Score**
Al Muallem et al. [21]				O		****
Barritt et al. [13]				O		**
Bell et al. [18]				O		****
Boo et al. [16]				O		****
Bruylands et al. [10]			O			**
Choi et al. [22]				O		**
Coffey et al. [23]				O		*****
Hampe et al. [14]			O			**
Jamieson et al. [11]					O	*****
Jang et al. [15]				O		*****
Liu et al. [12]				O		*****
Lucas et al. [24]				O		*
McCamley et al. [25]				O		*
Montagna et al. [19]		O				**
Thoroddsen et al. [26]				O		**
Yadav et al. [17]				O		*****
Zargaran et al. [20]				O		***

### Study Risk of Bias Assessment

The Mixed Methods Appraisal Tool (MMAT) (Version 2018) proposed by Hong et al. was used to assess the quality of the included studies [9]. MMAT is a specially designed tool that can be used for assessing the quality of different study types in the same review including qualitative, quantitative, and mixed methods studies. The assessment was conducted independently by FW and GF with discrepancies discussed within the research team (FW, GF, UK). Following the recommendations for reporting the results of the MMAT (Version 2018) the studies were rated on a scale of zero to five stars. Each of the five conditions that was met scored as one, an unclear or unmet condition scored as zero. Studies with low quality were not excluded for this review, but quality of included studies was presented and a possible risk of bias discussed on basis of the MMAT rating.

## Results

**Fig. 1:**
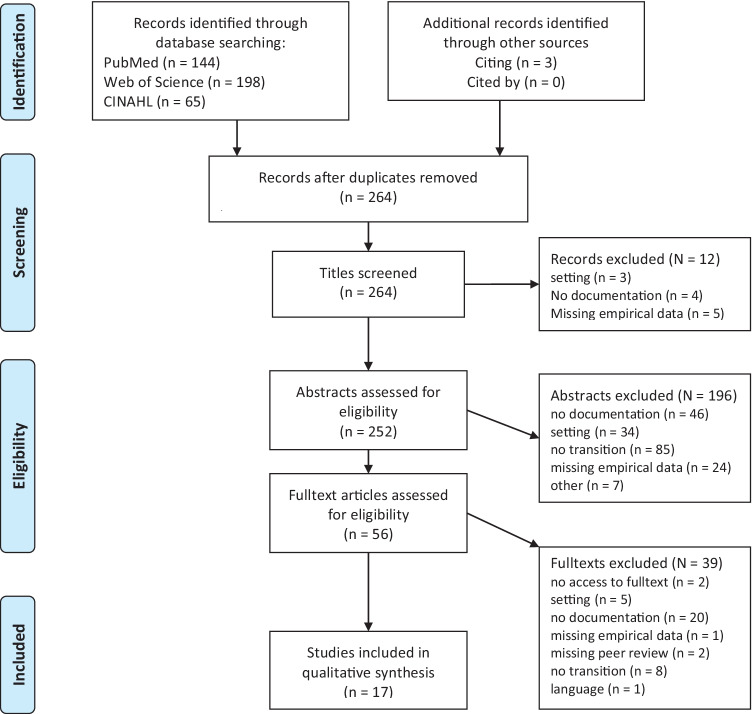
PRISMA 2009 Flow diagram

The study selection process and the reasons for excluding studies are depicted in Fig. 1. The database search resulted in 261 studies after duplicates were removed, plus three studies that were identified through a backward search of the records of the included studies [10–12]. 12 studies were excluded after title screening, 196 studies were excluded after abstracts were assessed for eligibility, and 39 studies were excluded after full texts were assessed for eligibility. The remaining 17 studies were included in this systematic review.

All included studies examine the documentation by performing a document analysis with comparison of the paper-based patient records and EPRs. Due to the hospital setting and the explicit exclusion of the outpatient setting, this concerns only the hospital's internal documentation in the patient records. Although the hospital setting was an inclusion criterion, the hospital setting still varies. There are differences in specialty (e.g., burn unit or orthopedic surgical ward) [13, 14], size (e.g., 700 beds or 1,200 beds) [15, 16], academical teaching activity, and one hospital which was not further specified [17]. Derived from that, all included studies investigate the documentation through the lens of a certain use case like for example operation reports or discharge instructions [14, 18]. The number of analyzed records varies from a minimum of 40 records (20 paper records vs. 20 electronic records) [19] to a maximum of 20,848 records (9,236 paper records vs. 11,612 electronic records) [20]. Except for Jamieson et al. who followed a prospective study design, all other studies evaluated the patient records retrospectively [11]. Only Montagna et al. followed a mixed methods approach, also investigating qualitative aspects such as the structure of the patient record in general or the format of the documentation in particular [19]. See Table 2 for detailed characteristics of all included studies.

The most commonly analyzed outcomes were completeness [15, 17, 20, 21, 23, 25, 26], guideline adherence [13, 14, 18, 22], and volume of documentation [11, 16, 17, 19]. Of all included studies, 11 of 17 proved a positive effect of the introduction of the EPR on documentation. Six of 17 showed a mixed effect with positive and negative changes, or no changes while no study showed an exclusively negative effect. Table 3 gives an overview of the analyzed outcomes, the key results of all included studies and whether a positive (+), negative (-), mixed (~) effect was measured. If the authors specified a p-value, it is indicated in the table. See Online Resource 4 for detailed summaries of all included studies.

MMAT was used to assess the individual risk of bias in the included studies and to rate their quality based on questions like “Are there complete outcome data?”. The two screening questions whether the study is an empirical one were fulfilled in all cases except one study [14]. That study fulfilled only one of the two screening questions with the second remaining unclear. Nevertheless, all studies were evaluated in terms of their quality. In Table 4, the final MMAT score of all included studies is depicted with a maximum of five stars. The detailed rating of all individual conditions is accessible in the appendix (Online Resource 5) which might be important since many conditions may not necessarily be unmet but remain unclear. Jamieson et al. and Liu and Edye used the QNOTE-instrument to measure their outcome [11, 12, 27], while Bruylands et al. used the Q-DIO-instrument [10, 28]. All other studies did not use any validated instrument to measure their outcomes. Moreover, several studies did not define their outcomes [16, 19, 24], or did so only superficially [21]. None of the studies followed a theoretical framework.

## Discussion

The database search identified 264 studies of which 17 met the inclusion criteria. The majority of those showed improved documentation after the introduction of the EPR. Although none of the studies followed a theoretical framework, there are certainly several more general frameworks that might have suited after an adaption to the topic. A framework for data quality in medical research was presented [8], originally targeting registry data and cohort studies. This framework classifies a total of 51 items into the quality model according to Donabedian [29], with the underlying dimensions of structure, process, and outcome quality that also fits to the present research question. This means, for example, that the outcome “standardized nursing language” could be assigned to the framework’s item "values from standards" (proportion of values that correspond to terms from controlled vocabularies) and thus be assigned to the dimension structural quality. The classification of all outcomes shows that five out of 17 studies have examined structure quality and 13 out of 17 studies outcome quality. The used instruments were not classified as they attempted to cover multiple dimensions [27, 28].

EPRs provide the possibility to automatically fill fields with information that are collected from other digital sources. This was seen in the study by Jang et al. where electronic documentation significantly improved only the automatically documented items but not the manually documented items [15]. EPRs also provide mandatory fields that need to be filled before the record can be closed. Zargaran et al. assumed that higher rates of completeness which they found were mainly reached with mandatory entries in the EPR before the record can be closed [20]. Depending on the mechanism that determines the change in documentation, the literature shows different implications for practitioners. On the one hand, increased documentation effort is conceivable through the use of features such as pop-ups, mandatory fields, etc. On the other hand, there might be improved documentation with the same or even reduced documentation effort due to automatically filled fields and optimized layout [30]. Montagna et al. also described a general change in documentation format from a continuous text towards a clear list of events showing that the introduction of the EPR is also a possibility to shape the structure of documentation [19]. This gives the opportunity to involve practitioners, as they have important insight into how to reduce documentation burdens, as a recent study showed [31]. Overall, the EPR appears to improve documentation while it remains unclear whether this change will come at the cost of an additional burden on practitioners.

When talking about improved documentation, the interpretation of the presented results and outcomes is often ambiguous. For example, it is not clarified, whether the outcome volume of documentation evaluates length of documentation only or also takes information density into account. Therefore, a lengthening documentation is not necessarily to be evaluated negatively, if at the same time completeness increases and vice versa. Moreover, regarding the frequently analyzed outcome guideline adherence, it remains unclear whether the improvements are due to a mere change in documentation or whether the actual treatment has changed due to the introduction of the EPR and is more guideline-compliant thereafter. This could be the case if the EPR conveys guideline information or offers clinical decision support based on guidelines or care might have delivered but was not documented before the introduction and is now forced to be documented with mandatory fields.

A challenge of this review was the heterogeneity of the setting, outcomes, and the lack of the outcome definitions in some studies. However, the differently shaped setting and variety of outcomes gives a wide overview of the different applications of the EPR and how documentation changes in different views. Moreover, except for Zargaran et al. from South Africa [20], which is an upper middle income country [32], no studies from low- or middle-income countries were found, making it challenging to compare or transfer the results into all healthcare systems worldwide. A common difficulty is also the probability of a present publication bias. Publication bias was not assessed in this review but since it is conceivable that the analyzation of records is carried out internally and published afterwards, the risk of negative effects not being published is probably high. The fact that none of the included studies showed an exclusively negative effect underlines a suspected publication bias. The results must be interpreted with caution, since the MMAT rating proved low scores in several studies, meaning that the methodological standards in those studies imply a high risk of bias. It is important to highlight that of the five studies with the maximum MMAT score, implying only a small risk of bias, Jamieson et al., Yadav et al. and Coffey et al. show only mixed effects [11, 17, 23] and Jang et al. only a partially positive effect [15]. On the other hand, in those studies with lower MMAT ratings, only one showed a mixed effect [19] while the remaining seven studies all proved a solely positive effect. This shows that all but one effect that were not solely positive were proved in the studies with high methodological standards. Therefore it has to be underlined that a bias in the studies with low MMAT scores should be considered. In the matter of evidence there is only one randomized controlled trial [11].

There are some limitations of the present review that must be stated. Although the searched databases were carefully selected based on their topic and range, important results in other databases may still have been missed. Moreover, only studies from the last ten years were included. Nevertheless, some studies might have addressed the topic of this review, which were published before 2010 and could still be valid today. This could be an important aspect, as some healthcare systems are already highly digitized and thereby a lot of research might have been conducted before 2010. On the other hand, the results of this review generate evidence regarding the analyzation of change in documentation through EPRs of the current state of the art.

Due to the ongoing digital transformation of the healthcare systems worldwide, it is expected that many hospitals will continue to implement new EPRs or adapt existing EPRs in the future. Each of these episodes of organizational change offers the opportunity to customize the structure of the record in terms of what is documented where and how. This results in the possibility of optimizing documentation regarding treatment quality or billing purposes on the one hand. On the other hand, documenting itself could be made as non-stressful as possible for the healthcare professionals. To make this process efficient, a systematic analysis of the change in documentation is essential. Healthcare professionals should use the existing validated instruments to produce comparable results. Also, future research should aim at developing further, more specific instruments to make it as easy as possible for practitioners to systematically collect data and publish results. This allows growing evidence on how to design documentation in the best way for all parties involved.

## Supplementary Information

Below is the link to the electronic supplementary material.

Supplementary file1 (PDF 361 KB)

## Data Availability

All materials or data used are part of this manuscript or are accessible in the Online Resources/Additional Files. **Online Resource** 1 PRISMA checklist **Online Resource** 2 Search strategy **Online Resource** 3 Screening questionnaire **Online Resource** 4 Detailed summary of all included studies **Online Resource** 5 Mixed Methods Appraisal Tool Rating **Online Resource** 6 A priori protocol

## Abbreviations

ED: Emergency Department
EPR: Electronic Patient Record
MMAT: Mixed Methods Appraisal Tool
PRISMA: Preferred Reporting Items for Systematic Reviews and Meta-Analyses

## References

[1] Embi PJ, Weir C, Efthimiadis EN, Thielke SM, Hedeen AN, Hammond KW (2013). Computerized provider documentation: findings and implications of a multisite study of clinicians and administrators. Journal of the American Medical Informatics Association : JAMIA.

[2] Vos JFJ, Boonstra A, Kooistra A, Seelen M, van Offenbeek M (2020). The influence of electronic health record use on collaboration among medical specialties. BMC health services research.

[3] Gesner E, Gazarian P, Dykes P (2019) The burden and burnout in documenting patient care: An integrative literature review MEDINFO 2019: Health and wellbeing e-networks for all. IOS Press, S 1194–1198 10.3233/SHTI190415 31438114

[4] Dick RS, Steen EB, Detme DE (1997). Computer-based Patient Record: An Essential Technology for Health Care.

[5] Gunningberg L, Lindholm C, PhD MC, PhD P-OS (2000). The development of pressure ulcers in patients with hip fractures: inadequate nursing documentation is still a problem. J Adv Nurs.

[6] Bååth C, Hall-Lord M-L, Johansson I, Wilde Larsson B (2007). Nursing assessment documentation and care of hip fracture patients’ skin. Journal of Orthopaedic Nursing.

[7] Page MJ, McKenzie JE, Bossuyt PM, Boutron I, Hoffmann TC, Mulrow CD, Shamseer L, Tetzlaff JM, Akl EA, Brennan SE, Chou R, Glanville J, Grimshaw JM, Hróbjartsson A, Lalu MM, Li T, Loder EW, Mayo-Wilson E, McDonald S, McGuinness LA, Stewart LA, Thomas J, Tricco AC, Welch VA, Whiting P, Moher D (2021). The PRISMA 2020 statement: an updated guideline for reporting systematic reviews. BMJ (Clinical research ed.).

[8] Nonnemacher M, Nasseh D, Stausberg J (2014) Datenqualität in der medizinischen Forschung. Leitlinie zum adaptiven Management von Datenqualität in Kohortenstudien und Registern, 2. Aufl. Schriftenreihe der TMF - Technologie- und Methodenplattform für die Vernetzte Medizinische Forschung e.V, Band 4. Medizinisch Wissenschaftliche Verlagsgesellschaft, Berlin

[9] Hong QN, Pluye P, Fàbregues S, Bartlett G, Boardman F, Cargo M, Dagenais P, Gagnon M-P, Griffiths F, Nicolau B (2018). Mixed methods appraisal tool (MMAT), version 2018. Registration of copyright.

[10] Bruylands M, Paans W, Hediger H, Müller-Staub M (2013). Effects on the quality of the nursing care process through an educational program and the use of electronic nursing documentation. International journal of nursing knowledge.

[11] Jamieson T, Ailon J, Chien V, Mourad O (2017). An electronic documentation system improves the quality of admission notes: a randomized trial. Journal of the American Medical Informatics Association : JAMIA.

[12] Liu ZY, Edye M (2020). Implementation of electronic health records systems in surgical units and its impact on performance. ANZ Journal of Surgery.

[13] Barritt AW, Clark L, Cohen AMM, Hosangadi-Jayedev N, Gibb PA (2010). Improving the quality of procedure-specific operation reports in orthopaedic surgery. Annals of the Royal College of Surgeons of England.

[14] Hampe HM, Keeling T, Fontana M, Balcik D (2017). Impacting Care and Treatment of the Burn Patient Conversion to Electronic Documentation. Critical care nursing quarterly.

[15] Jang J, Yu SH, Kim C-B, Moon Y, Kim S (2013). The effects of an electronic medical record on the completeness of documentation in the anesthesia record. International journal of medical informatics.

[16] Boo Y, Noh YA, Kim M-G, Kim S (2012). A study of the difference in volume of information in chief complaint and present illness between electronic and paper medical records. Health information management : journal of the Health Information Management Association of Australia.

[17] Yadav S, Kazanji N, K C N, Paudel S, Falatko J, Shoichet S, Maddens M, Barnes MA,  (2017). Comparison of accuracy of physical examination findings in initial progress notes between paper charts and a newly implemented electronic health record. Journal of the American Medical Informatics Association : JAMIA.

[18] Bell EJ, Takhar SS, Beloff JR, Schuur JD, Landman AB (2013). Information Technology Improves Emergency Department Patient Discharge Instructions Completeness and Performance on a National Quality Measure A Quasi-Experimental Study. Applied clinical informatics.

[19] Montagna S, Croatti A, Ricci A, Agnoletti V, Albarello V, Gamberini E (2020). Real-time tracking and documentation in trauma management. Health Informatics Journal.

[20] Zargaran E, Spence R, Adolph L, Nicol A, Schuurman N, Navsaria P, Ramsey D, Hameed SM (2018). Association Between Real-time Electronic Injury Surveillance Applications and Clinical Documentation and Data Acquisition in a South African Trauma Center. JAMA surgery.

[21] Al Muallem Y, Al Dogether M, Househ M, Saddik B (2017). Auditing The Completeness and Legibility of Computerized Radiological Request Forms. Journal of medical systems.

[22] Choi CK, Saberito D, Tyagaraj C, Tyagaraj K (2014). Organizational Performance and Regulatory Compliance as Measured by Clinical Pertinence Indicators Before and After Implementation of Anesthesia Information Management System (AIMS). Journal of medical systems.

[23] Coffey C, Wurster LA, Groner J, Hoffman J, Hendren V, Nuss K, Haley K, Gerberick J, Malehorn B, Covert J (2015). A Comparison of Paper Documentation to Electronic Documentation For Trauma Resuscitations at a Level I Pediatric Trauma Center. Journal of emergency nursing.

[24] Lucas B, Schladitz P, Schirrmeister W, Pliske G, Walcher F, Kulla M, Brammen D (2019). The way from pen and paper to electronic documentation in a German emergency department. BMC health services research.

[25] Mccamley J, Vivant A, Edirippulige S (2019). Dietetics in the digital age: The impact of an electronic medical record on a tertiary hospital dietetic department. Nutrition & Dietetics.

[26] Thoroddsen A, Ehnfors M, Ehrenberg A (2011). Content and completeness of care plans after implementation of standardized nursing terminologies and computerized records. Computers, informatics, nursing : CIN.

[27] Burke HB, Hoang A, Becher D, Fontelo P, Liu F, Stephens M, Pangaro LN, Sessums LL, O'Malley P, Baxi NS (2014). QNOTE: an instrument for measuring the quality of EHR clinical notes. Journal of the American Medical Informatics Association : JAMIA.

[28] Müller-Staub M, Lunney M, Lavin MA, Needham I, Odenbreit M, van Achterberg T (2008). Testing the Q-DIO as an instrument to measure the documented quality of nursing diagnoses, interventions, and outcomes. International journal of nursing terminologies and classifications : the official journal of NANDA International.

[29] Donabedian A (1988). The quality of care: how can it be assessed?. JAMA.

[30] Ommaya AK, Cipriano PF, Hoyt DB, Horvath KA, Tang P, Paz HL, DeFrancesco MS, Hingle ST, Butler S, Sinsky CA (2018) Care-centered clinical documentation in the digital environment: solutions to alleviate burnout. NAM Perspectives 8(1). 10.31478/201801c

[31] Sieja A, Markley K, Pell J, Gonzalez C, Redig B, Kneeland P, Lin C-T (2019). Optimization Sprints: Improving Clinician Satisfaction and Teamwork by Rapidly Reducing Electronic Health Record Burden. Mayo Clinic Proceedings.

[32] The World Bank Group (2021) World bank country and lending groups. https://datahelpdesk.worldbank.org/knowledgebase/articles/906519-world-bank-country-and-lending-groups. Accessed: 05 Nov 2021

