# Supplementary material for: The Analyzation of Change in Documentation due to the Introduction of Electronic Patient Records in Hospitals—A Systematic Review
Source: J Med Syst. 2022 Jul 4;46(8):54. doi: 10.1007/s10916-022-01840-0 (PMC9252957; doi:10.1007/s10916-022-01840-0)
Supplement: Supplementary file 1 — Supplementary file1 (PDF 361 KB) [file 10916_2022_1840_MOESM1_ESM.pdf]

Additional File 1: PRISMA 2020 Checklist

The Analyzation of Change in Documentation due to the Introduction of Electronic Patient Records in Hospitals - A Systematic Review

Authors
Florian Wurster[1]
Garret Fütterer[1]
Marina Beckmann[1]
Kerstin Dittmer[1]
Julia Jaschke[2]
Juliane Köberlein-Neu[2]
Mi-Ran Okumu[1]
Carsten Rusniok[1]
Holger Pfaff[1]
Ute Karbach[1]

Affiliations
[1] University of Cologne, Faculty of Human Sciences & Faculty of Medicine and University Hospital Cologne, Institute of Medical Sociology, Health Services Research, and Rehabilitation Science, Germany
[2] University of Wuppertal, Center for Health Economics and Health Services Research, Germany

Correspondence
Florian Wurster, M.Sc.
+49 221 478-97116
florian.wurster@uni-koeln.de
Eupener Str. 129
50933 Cologne, Germany

Additional File 1: PRISMA 2020 Checklist

PRISMA 2020 Checklist

Section and Topic	Item #	Checklist item	Location where item is reported
TITLE			
Title	1	Identify the report as a systematic review.	p. 3
ABSTRACT			
Abstract	2	See the PRISMA 2020 for Abstracts checklist.	p. 2
INTRODUCTION			
Rationale	3	Describe the rationale for the review in the context of existing knowledge.	p. 3
Objectives	4	Provide an explicit statement of the objective(s) or question(s) the review addresses.	p. 3
METHODS			
Eligibility criteria	5	Specify the inclusion and exclusion criteria for the review and how studies were grouped for the syntheses.	p. 3, table 1
Information sources	6	Specify all databases, registers, websites, organisations, reference lists and other sources searched or consulted to identify studies. Specify the date when each source was last searched or consulted.	p. 3
Search strategy	7	Present the full search strategies for all databases, registers and websites, including any filters and limits used.	p. 3
Selection process	8	Specify the methods used to decide whether a study met the inclusion criteria of the review, including how many reviewers screened each record and each report retrieved, whether they worked independently, and if applicable, details of automation tools used in the process.	p. 3
Data collection process	9	Specify the methods used to collect data from reports, including how many reviewers collected data from each report, whether they worked independently, any processes for obtaining or confirming data from study investigators, and if applicable, details of automation tools used in the process.	p. 4
Data items	10a	List and define all outcomes for which data were sought. Specify whether all results that were compatible with each outcome domain in each study were sought (e.g. for all measures, time points, analyses), and if not, the methods used to decide which results to collect.	p. 6, table 3
	10b	List and define all other variables for which data were sought (e.g. participant and intervention characteristics, funding sources). Describe any assumptions made about any missing or unclear information.	n.a.
Study risk of bias assessment	11	Specify the methods used to assess risk of bias in the included studies, including details of the tool(s) used, how many reviewers assessed each study and whether they worked independently, and if applicable, details of automation tools used in the process.	p. 4
Effect measures	12	Specify for each outcome the effect measure(s) (e.g. risk ratio, mean difference) used in the synthesis or presentation of results.	p. 7, table 4

Synthesis methods	13a	Describe the processes used to decide which studies were eligible for each synthesis (e.g. tabulating the study intervention characteristics and comparing against the planned groups for each synthesis (item #5)).	p. 4
	13b	Describe any methods required to prepare the data for presentation or synthesis, such as handling of missing summary statistics, or data conversions.	n.a.
	13c	Describe any methods used to tabulate or visually display results of individual studies and syntheses.	p. 7, table 4
	13d	Describe any methods used to synthesize results and provide a rationale for the choice(s). If meta-analysis was performed, describe the model(s), method(s) to identify the presence and extent of statistical heterogeneity, and software package(s) used.	p. 4
	13e	Describe any methods used to explore possible causes of heterogeneity among study results (e.g. subgroup analysis, meta-regression).	p. 4
	13f	Describe any sensitivity analyses conducted to assess robustness of the synthesized results.	n.a.
Reporting bias assessment	14	Describe any methods used to assess risk of bias due to missing results in a synthesis (arising from reporting biases).	p. 4
Certainty assessment	15	Describe any methods used to assess certainty (or confidence) in the body of evidence for an outcome.	p. 7
RESULTS			
Study selection	16a	Describe the results of the search and selection process, from the number of records identified in the search to the number of studies included in the review, ideally using a flow diagram.	Figure F
	16b	Cite studies that might appear to meet the inclusion criteria, but which were excluded, and explain why they were excluded.	p. 4
Study characteristics	17	Cite each included study and present its characteristics.	Additional File 2
Risk of bias in studies	18	Present assessments of risk of bias for each included study.	p. 7, table 4
Results of individual studies	19	For all outcomes, present, for each study: (a) summary statistics for each group (where appropriate) and (b) an effect estimate and its precision (e.g. confidence/credible interval), ideally using structured tables or plots.	Additional File 2
Results of syntheses	20a	For each synthesis, briefly summarise the characteristics and risk of bias among contributing studies.	p. 7, table 4
	20b	Present results of all statistical syntheses conducted. If meta-analysis was done, present for each the summary estimate and its precision (e.g. confidence/credible interval) and measures of statistical heterogeneity. If comparing groups, describe the direction of the effect.	n.a
	20c	Present results of all investigations of possible causes of heterogeneity among study results.	p. 4
	20d	Present results of all sensitivity analyses conducted to assess the robustness of the synthesized results.	n.a.
Reporting biases	21	Present assessments of risk of bias due to missing results (arising from reporting biases) for each synthesis assessed.	p. 7
Certainty of evidence	22	Present assessments of certainty (or confidence) in the body of evidence for each outcome assessed.	p. 7

DISCUSSION			
Discussion	23a	Provide a general interpretation of the results in the context of other evidence.	p. 7
	23b	Discuss any limitations of the evidence included in the review.	p. 7
	23c	Discuss any limitations of the review processes used.	p. 8
	23d	Discuss implications of the results for practice, policy, and future research.	p. 8
OTHER INFORMATION			
Registration and protocol	24a	Provide registration information for the review, including register name and registration number, or state that the review was not registered.	p. 9
	24b	Indicate where the review protocol can be accessed, or state that a protocol was not prepared.	p. 9
	24c	Describe and explain any amendments to information provided at registration or in the protocol.	--
Support	25	Describe sources of financial or non-financial support for the review, and the role of the funders or sponsors in the review.	p.p. 2, 4 & 9
Competing interests	26	Declare any competing interests of review authors.	p. 9
Availability of data, code and other materials	27	Report which of the following are publicly available and where they can be found: template data collection forms; data extracted from included studies; data used for all analyses; analytic code; any other materials used in the review.	p. 9

From: Page MJ, McKenzie JE, Bossuyt PM, et al. The PRISMA 2020 statement: an updated guideline for reporting systematic reviews. BMJ 2021;372:n71. doi:10.1136/bmj.n71 For more information, visit: www.prisma-statement.org.

Additional File 2: Search of Databases including Results and Dates

The Analyzation of Change in Documentation due to the Introduction of Electronic Patient Records in Hospitals - A Systematic Review

Authors
Florian Wurster[1]
Garret Fütterer[1]
Marina Beckmann[1]
Kerstin Dittmer[1]
Julia Jaschke[2]
Juliane Köberlein-Neu[2]
Mi-Ran Okumu[1]
Carsten Rusniok[1]
Holger Pfaff[1]
Ute Karbach[1]

Affiliations
[1] University of Cologne, Faculty of Human Sciences & Faculty of Medicine and University Hospital Cologne, Institute of Medical Sociology, Health Services Research, and Rehabilitation Science, Germany
[2] University of Wuppertal, Center for Health Economics and Health Services Research, Germany

Correspondence
Florian Wurster, M.Sc.
+49 221 478-97116
florian.wurster@uni-koeln.de
Eupener Str. 129
50933 Cologne, Germany

Additional File 2: Search of Databases including Results and Dates

Electronic database	First component “implementation”	Boolean operator	Second component “electronic health record”	Boolean operator	third component “paperbased”	Boolean operator	fourth component "documentation"	Boolean operator	fifth component "clinic"	Results	Date	Filter
**Pubmed, PubmedCentral, MEDLINE via Pubmed**	((((((Implement*[Title/Abstract]) OR (adopt*[Title/Abstract])) OR (introduc*[Title/Abstract])) OR (transition*[Title/Abstract])) OR (Launch*[Title/Abstract])) OR (establish*[Title/Abstract])) OR ("implementation science"[MeSH Major Topic])	AND	((((((((((((((((((((((("elektronische fallakte"[Title/Abstract]) OR ("electronic health record*"[Title/Abstract])) OR ("ehr"[Title/Abstract])) OR ("electronic medical record*"[Title/Abstract])) OR ("emr"[Title/Abstract])) OR ("electronic medical health record*"[Title/Abstract])) OR ("electronic medical patient record*"[Title/Abstract])) OR ("electronic medical records system"[Title/Abstract])) OR ("emrs"[Title/Abstract])) OR ("electronic medical note*"[Title/Abstract])) OR ("electronic medical file*"[Title/Abstract])) OR ("electronic patient record*"[Title/Abstract])) OR ("epr"[Title/Abstract])) OR ("electronic patient chart*"[Title/Abstract])) OR ("electronic clinical note*"[Title/Abstract])) OR ("electronic clinical record*"[Title/Abstract])) OR (electronic health records[MeSH Terms])) OR (electronic health record[MeSH Terms])) OR (computerized medical records system[MeSH Terms])) OR (computerized medical records systems[MeSH Terms])) OR ("computerized medical record*"[Title/Abstract])) OR ("computerized health record*"[Title/Abstract])) OR ("computerized patient record*"[Title/Abstract)	AND	((((((paper-based[Title/Abstract]) OR (paper based[Title/Abstract])) OR (on-paper[Title/Abstract])) OR (hardcopy[Title/Abstract])) OR (handwritten[Title/Abstract])) OR (in writing[Title/Abstract])) OR ("paper"[Title/Abstract])	AND	("documentation"[Title/Abstract]) OR ("documentation"[MeSH Major Topic])	AND	((((((((((Krankenh*[Title/Abstract]) OR (Klinik*[Title/Abstract])) OR (spital[Title/Abstract])) OR ("hospital"[Title/Abstract])) OR (hospitals[Title/Abstract])) OR ("clinic"[Title/Abstract])) OR ("clinics"[Title/Abstract])) OR (ward*[Title/Abstract])) OR (Infirmar*[Title/Abstract])) OR ("medical center*"[Title/Abstract])) OR ("health center*"[Title/Abstract])	**144**	08.01.2021	2010-2020
**CINAHL via EBSCOhost**	TI ((implement*) OR (adopt*) OR (introduc*) OR (transition*) OR (launch*) OR (establish*)) OR AB ((implement*) OR (adopt*) OR (introduc*) OR (transition*) OR (launch*) OR (establish*))	AND	(TI (("electronic health record") OR ("ELECTRONIC HEALTH RECORDS") OR (“computerized medical record”) OR (“computerized medical records”) OR (“computerized health record”) OR (“computerized health records”) OR (“computerized patient record”) OR (“computerized patient records”) OR (“computerised medical record”) OR (computerised medical records”) OR (“computerised health record”) OR (computerised health records”) OR (“computerised patient record”) OR (“computerised patient records”) OR ("ELECTRONIC MEDICAL RECORD") OR ("ELECTRONIC MEDICAL RECORDS") OR ("EMR") OR ("ELECTRONIC MEDICAL HEALTH RECORD") OR ("ELECTRONIC MEDICAL HEALTH RECORDS") OR ("ELECTRONIC MEDICAL PATIENT RECORD") OR ("ELECTRONIC MEDICAL PATIENT RECORDS") OR ("ELECTRONIC MEDICAL RECORDS SYSTEM") OR ("EMRS") OR	AND	TI (("paper-based") OR ("paper based") OR (paperbased) OR ("on-paper") OR (hardcopy) OR (handwritten) OR (in writing) OR ("paper")) OR AB (("paper-based") OR ("paper based") OR (paperbased)	AND	TI (documentation) OR AB (documentation)	AND	TI ((hospital) OR (clinic) OR (ward) OR (infirmary) OR ("medical center") OR ("medical centers") OR ("health center")) OR AB ((hospital) OR (clinic) OR (ward) OR (infirmary) OR ("medical center") OR ("medical centers") OR ("health center"))	**65**	08.01.2021	2010-2020

			("ELECTRONIC MEDICAL NOTE") OR ("ELECTRONIC MEDICAL NOTES") OR ("ELECTRONIC MEDICAL FILE") OR ("ELECTRONIC MEDICAL FILES") OR ("ELECTRONIC PATIENT RECORD") OR ("ELECTRONIC PATIENT RECORDS") OR ("EPR") OR ("ELECTRONIC PATIENT CHART") OR ("ELECTRONIC PATIENT CHARTS") OR ("ELECTRONIC CLINICAL NOTE") OR ("ELECTRONIC CLINICAL NOTES") OR ("ELECTRONIC CLINICAL RECORD") OR ("ELECTRONIC CLINICAL RECORDS")) OR AB (("electronic health record") OR ("ELECTRONIC HEALTH RECORDS") OR ("ELECTRONIC MEDICAL RECORD") OR ("ELECTRONIC MEDICAL RECORDS") OR ("EMR") OR ("ELECTRONIC MEDICAL HEALTH RECORD") OR ("ELECTRONIC MEDICAL HEALTH RECORDS") OR ("ELECTRONIC MEDICAL PATIENT RECORD") OR ("ELECTRONIC MEDICAL PATIENT RECORDS") OR ("ELECTRONIC MEDICAL RECORDS SYSTEM") OR ("EMRS") OR ("ELECTRONIC MEDICAL NOTE") OR ("ELECTRONIC MEDICAL NOTES") OR ("ELECTRONIC MEDICAL FILE") OR ("ELECTRONIC MEDICAL FILES") OR ("ELECTRONIC PATIENT RECORD") OR ("ELECTRONIC PATIENT RECORDS") OR ("EPR") OR ("ELECTRONIC PATIENT CHART") OR ("ELECTRONIC PATIENT CHARTS") OR ("ELECTRONIC CLINICAL NOTE") OR ("ELECTRONIC CLINICAL NOTES") OR ("ELECTRONIC CLINICAL RECORD") OR ("ELECTRONIC CLINICAL RECORDS")))		OR ("on-paper") OR (hardcopy) OR (handwritten) OR (in writing) OR ("paper"))							
**Web of Science Core Collection**	TS=(implement* OR adopt* OR introduc* OR transition* OR Launch* OR establish*)	AND	TS=(elektronische fallakte OR electronic health record$ OR ehr OR electronic medical record$ OR emr OR computei?ed health redord$ OR computeri?ed medical record$ OR computeri?ed patient record$ OR electronic medical health record$ OR electronic medical patient record$ OR electronic medical records system OR emrs OR electronic medical Note$ OR electronic medical file$ OR electronic patient record$ OR epr OR electronic patient chart$ OR electronic clinical note OR electronic clinical record$)	AND	TS=(paper-based OR paperbased OR paper based OR on-paper OR hardcopy OR handwritten OR "in writing" OR "paper")	AND	TS=(documentation)	AND	TS=(krankenh* OR Klinik* OR spital OR hospital$ OR clinic$ OR ward$ OR Infirmar* OR medical center$ OR medical center$ OR health center$)	**198**	08.01.2021	2010-2020
**PDQ Evidence**	(title:(adopt*) OR abstract:(adopt*)) OR (title:(introduc*) OR abstract:(introduc	AND	(title:("electronic health record") OR abstract:("electronic health record")) OR (title:("electronic health records") OR abstract:("electronic health records")) OR (title:("ehr") OR abstract:("ehr")) OR (title:("electronic medical record") OR abstract:("electronic medical record")) OR (title:("electronic medical records") OR abstract:("electronic medical records")) OR (title:("computerized medical records") OR	AND	(title:(paper-based) OR abstract:(paper-based)) OR (title:(paperbased) OR abstract:(paper	AND	(title:(documentation) OR abstract: (documentation))	AND	(title:("hospital") OR abstract:("hospital")) OR (title:("hospitals") OR abstract:("hospitals	**0**	08.01.2021	2010-2020

	)) OR (title:(transition) OR abstract:(transition*)) OR (title:(launch*) OR abstract:(launch*)) OR (title:(establish*) OR abstract:(establish*))		abstract:("computerized medical records")) OR (title:("computerized medical record") OR abstract:("computerized medical record")) OR (title:("computerized health record") OR abstract:("computerized health record")) OR (title:("computerized health records") OR abstract:("computerized health records")) OR (title:("computerized patient record") OR abstract:("computerized patient record")) OR (title:("computerized patient records") OR abstract:("computerized patient records")) OR (title:("computerised health record") OR abstract:("computerised health record")) OR (title:("computerised health records") OR abstract:("computerised health records")) OR (title:("computerised medical record") OR abstract:("computerised medical record")) OR (title:("computerised medical records") OR abstract:("computerised medical records")) OR (title:("computerised patient record") OR abstract:("computerised patient record")) OR (title:("computerised patient records") OR abstract:("computerised patient records")) OR (title:("emr") OR abstract:("emr")) OR (title:("electronic medical health record") OR abstract:("electronic medical health record")) OR (title:("electronic medical health records") OR abstract:("electronic medical health records")) OR (title:("electronic medical patient record") OR abstract:("electronic medical patient record")) OR (title:("electronical medical patient records") OR abstract:("electronical medical patient records")) OR (title:("electronical medical records system") OR abstract:("electronical medical records system")) OR (title:("emrs") OR abstract:("emrs")) OR (title:("electronic medical note") OR abstract:("electronic medical note")) OR (title:("electronic medical notes") OR abstract:("electronic medical notes")) OR (title:("electronic medical file") OR abstract:("electronic medical file")) OR (title:("electronic medical files") OR abstract:("electronic medical files")) OR (title:("electronic patient record") OR abstract:("electronic patient record")) OR (title:("electronic patient records") OR abstract:("electronic patient records")) OR (title:("epr") OR abstract:("epr")) OR (title:("electronic patient chart") OR abstract:("electronic patient chart")) OR (title:("electronic patient charts") OR abstract:("electronic patient charts")) OR (title:("electronic clinical note") OR abstract:("electronic clinical note")) OR (title:("electronic clinical notes") OR abstract:("electronic clinical notes")) OR (title:("electronic clinical record") OR abstract:("electronic		based)) OR (title:(paper based) OR abstract:(paper based)) OR (title:(on-paper) OR abstract:(on-paper)) OR (title:(hardcopy) OR abstract:(hardcopy)) OR (title:(handwritten) OR abstract:(handwritten)) OR (title:(in writing) OR abstract:(in writing)) OR (title:("paper") OR abstract:("paper"))				")) OR (title:("clinic") OR abstract:("clinic")) OR (title:("clinics") OR abstract:("clinics")) OR (title:(ward*) OR abstract:(ward*)) OR (title:(infirmar*) OR abstract:(infirmar*)) OR (title:("medical center") OR abstract:("medical center")) OR (title:("medcal centers") OR abstract:("medcal centers")) OR (title:("health center") OR abstract:("health center")) OR (title:("health centers") OR abstract:("health centers"))			

			clinical record")) OR (title:("electronic clinical records") OR abstract:("electronic clinical records"))									

Additional File 3: Inclusion criteria check list

The Analyzation of Change in Documentation due to the Introduction of Electronic Patient Records in Hospitals - A Systematic Review

Authors
Florian Wurster[1]
Garret Fütterer[1]
Marina Beckmann[1]
Kerstin Dittmer[1]
Julia Jaschke[2]
Juliane Köberlein-Neu[2]
Mi-Ran Okumu[1]
Carsten Rusniok[1]
Holger Pfaff[1]
Ute Karbach[1]

Affiliations
[1] University of Cologne, Faculty of Human Sciences & Faculty of Medicine and University Hospital Cologne, Institute of Medical Sociology, Health Services Research, and Rehabilitation Science, Germany
[2] University of Wuppertal, Center for Health Economics and Health Services Research, Germany

Correspondence
Florian Wurster, M.Sc.
+49 221 478-97116
florian.wurster@uni-koeln.de
Eupener Str. 129
50933 Cologne, Germany

Additional File 3: Inclusion criteria check list

Source:

……

………

1. Is the study written in English or German language?				
Yes ☐	on to question 2	No	☐	exclusion
2. Is documentation part of the object studied?				
Yes ☐	on to question 3	No	☐	exclusion
3. Is the study set in a hospital setting?				
Yes ☐	on to question 4	No	☐	exclusion
4. Does the focus lie on the transition of paper-based to electronic documentation?				
Yes ☐	on to question 5	No	☐	exclusion
5. Was original empirical research conducted in the study?				
Yes ☐	inclusion	No	☐	on to question 6
6. Are original empirical research sources contained in the literature review also contained in the search results for this review?				
Yes ☐	exclusion		☐	Inclusion of source

Initials of reviewer: ……………………………………

Additional File 4: Summaries of Included Studies

The Analyzation of Change in Documentation due to the Introduction of Electronic Patient Records in Hospitals - A Systematic Review

Authors
Florian Wurster[1]
Garret Fütterer[1]
Marina Beckmann[1]
Kerstin Dittmer[1]
Julia Jaschke[2]
Juliane Köberlein-Neu[2]
Mi-Ran Okumu[1]
Carsten Rusniok[1]
Holger Pfaff[1]
Ute Karbach[1]

Affiliations
[1] University of Cologne, Faculty of Human Sciences & Faculty of Medicine and University Hospital Cologne, Institute of Medical Sociology, Health Services Research, and Rehabilitation Science, Germany
[2] University of Wuppertal, Center for Health Economics and Health Services Research, Germany

Correspondence
Florian Wurster, M.Sc.
+49 221 478-97116
florian.wurster@uni-koeln.de
Eupener Str. 129
50933 Cologne, Germany

Additional File 4: Summaries of Included Studies

Al Muallem et al. (2017) compared 456 medical imaging forms (228 paper records vs. 228 electronic records) from a radiology department at a military hospital in Saudi Arabia. They analyzed completeness and legibility and found electronic records to be significantly more complete (63·6% vs. 100%) ($p < 0·001$) and more legible (19·6% vs. 100%) ($p < 0·001$) than paper-based records.
Barritt et al. (2010) compared 80 operation reports for unilateral hip hemiarthroplasty (50 paper records vs. 30 electronic records) in an orthopaedic surgical ward in Great Britain. They analyzed the adherence to The Royal College of Surgeons of England (RCSE) guidelines for ideal documentation in operation reports, which was assessed using a 33-point scoring system. Paper-based records scored an average of 19·4 points (58·7%, 95% CI [56·6 – 60·8]) vs. 30·6 points (92·8%, 95% CI [91·0 – 94·6]) in electronic records, which was an improvement in every RCSE parameter and means a significant ($p < 0·01$) improvement of guideline adherence.
Bell et al. (2013) compared 300 discharge instructions (150 paper records vs. 150 electronic records) in an US emergency department. They analyzed the adherence to the Outpatient Measure 19 (OP-19) that was designed by the Centers for Medicare and Medicaid Services (CMS) to evaluate the quality of discharge instructions from emergency departments. Overall OP-19 compliance was 46·7% in paper-based discharge instructions vs. 97·3% in electronic discharge instructions, meaning that electronic discharge instructions were twice as likely to achieve overall OP-19 compliance compared to the paper-based format (RR: 2·09, 95% CI [1·75 – 2·48]) ($p < 0·05$).
Boo et al. (2012) compared the documentation of chief complaint and present illness in 2,281 documents (1,159 paper records vs. 1,122 electronic records). They analyzed the volume of information by word count. Due to the specific nature of the Korean language, “normalized bytes” were also counted. The volume of documented present illness decreased in the EPR when measured by number of words (25·95 ± 13·84 vs. 24·70 ± 14·16) ($p = 0·03$). The volume of documented present illness measured by normalized bytes and the volume of documented chief complaint did not decrease, neither measured by words, nor by normalized bytes.
Bruylands et al. (2013) compared the documentation of nursing diagnoses in 108 documents (3 groups of 36 records) in a Swiss general hospital. The first group originated one year after the introduction of nursing diagnoses, the second group two years later. Both were documenting in paper-based records. The third group originated three years after the introduction of the EPR. They analyzed the usage and frequency of standardized nursing terms with the Q-DIO-instrument (Müller-Staub et al., 2008) and found a ratio of 6·6 standardized nursing diagnoses in the EPR to 1·0 standardized nursing diagnose in the paper record. This is assumed to have arisen through autofill functions in the EPR. There are no further statistical values or tests presented.
Choi et al. (2014) compared 4,981 documents regarding pre-, peri-, or postoperative care (3,997 paper records vs. 984 electronic records) in an US academic teaching hospital. They analyzed the documentation of 14 certain clinical pertinence indicators, derived from multiple guidelines like Surgical Care Improvement Project (SCIP) or guidelines from the Centers for Medicare and Medicaid Services (CMS). They showed that overall adherence significantly increased (96·2% vs. 99·0%) ($p < 0·001$) in the EPR, mainly due to improvements in the documentation of used medications (75·6 – 92·3% vs. 98·5%) ($p < 0·001$) and documentation of physiological status, mental status, and postoperative pain level (sums as one indicator) (55·9 – 86·6% vs. 96·2%) ($p < 0·001$).
Coffey et al. (2015) compared 400 trauma resuscitation documents (200 paper records vs. 200 electronic records) from a US pediatric trauma center. They analyzed completeness of 11 elements, which were level of alert, time of alert activation, ABC assessment, cause of injury, arrival time in emergency department, treatment PTA by EMS, Volume of IV fluids PTA, arrival time of attending physician, documentation of vital signs, completion of ED IV fluids and disposition. Completeness of several elements improved in the EPR, particularly time of alert activation (85% vs. 100%) ($p < 0·001$), ABC assessment (88% vs. 94%) ($p < 0·05$), arrival time of attending physician (93·5% vs. 98%) ($p < 0·05$), completion of ED IV fluids (88% vs. 94%) ($p < 0·05$) and disposition (89·5% vs. 100%) ($p < 0·001$). In contrast, completeness of volume of IV fluids PTA documentation was higher in the paper-based record than in the EPR (100% vs. 68%) ($p < 0·001$). The remaining five elements did not show a significant change in documentation.
Hampe et al. (2017) compared the documentation in a specialized sheet for burn injuries at a burn unit of a tertiary hospital in the United States. They analyzed the adherence to the Lund Browder chart which is completed at admission and then every following Sunday and includes, e.g., wound size or degree of burning. The comparison of an uncertain number of records showed an increased adherence in the EPR (74% vs. 100%). There are no further statistical values or tests presented.
Jamieson et al. (2017) compared 42 admission notes (21 paper records vs. 21 electronic records) in an internal medicine ward of an academic teaching hospital in Canada. They randomized admitting teams to document either in paper-based or electronic records for two weeks and then changed the medium for two more weeks. This led to the existence of both, paper and electronic documentation, from the same person which was then randomly sampled and prepared for analyzation. To blind the evaluation, all notes were transcribed so that raters were not aware if the note was produced in paper-based or electronic documentation. Thereupon, quality of documentation was compared, using the QNOTE-instrument (Burke et al., 2014) with the highest possible score of 100. It showed significantly higher overall quality of documentation in the EPR (69 99% CI [61 – 77] vs. 90 99% CI [86 – 93]) ($p < 0·0001$) and higher quality in the free-text subsections History of Present Illness (78 99% CI [70 – 85] vs. 93

99% CI [89 – 98]) ($p < 0·0001$) and Impression and Plan (77 99% CI [70 – 84] vs. 89 99% CI [84 – 94]) (p = 0·001). Length of both free-text subsections were found to be higher in the EPR with History of Present Illness (92·4 99% CI [69·6 - 115·2] vs. 172·4 99% CI [122·7 - 222·0]) (p = 0·0001) and Impression and Plan (105·5 99% CI [77·2 - 133·7] vs. 140·4 99% CI [114·7 - 166·01]) ($p < 0·05$).

Jang et al. (2013) compared the anesthesia documentation in 250 documents (100 paper records vs. 150 electronic records) in a large Korean hospital. They analyzed completeness of documentation by assessing the presence of a total of 34 items. These were based solely on a checklist by Tsai & Bond (2008) but were modified according to standards e.g. defined by the Association of Nurse Anaesthetists of the Korean Nurses Association or the anesthesia records of American Association of Nurse Anaesthetists. They showed an overall increased completeness in the EPR (30·27 ± 3·02 vs. 31·34 ± 2·96) ($p < 0·01$). However, only those items improved that were automatically transferred into the EPR (11·08 ± 0·97 vs. 11·65 ± 0·71) ($p < 0·01$) but not those that were manually recorded.

Liu et al. (2020) compared 318 documents (98 paper records (group 1) vs. 107 electronic records (group 2, immediately after introduction) vs. 113 electronic records (group 3, 12 months after introduction)) of patients that had undergone appendicectomy. They used the QNOTE instrument (Burke et al., 2014) with the highest possible score of 100. They showed a significant improvement of 9 points (CI [4 – 9]) (p = 0·003) between group 1 and group 2 and a significant improvement of 8 points (CI [5 – 11]) (p = 0·001) between group 1 and group 3 but found no difference between group 2 and group 3.

Lucas et al. (2019) compared 10,891 standardized German Emergency Department Medical Records (GEDMR) (3,199 paper records vs. 2,910 electronic records (immediately after introduction) vs. 4,782 electronic records (6 months after introduction)) of the emergency departments trauma center in an academic teaching hospital in Germany. They analyzed the frequency of structured documentation in 20 information fields that included, among others, allergies and main diagnosis. The subject of what they called “structured documentation” was not further defined. However, they reported significantly ($p < 0·05$) increased structured documentation in all but two information fields (presenting complaints and discharge field) without giving detailed numbers or the specific differences between all three groups.

McCamley et al. (2019) analyzed documentation of nutritional data and dietetic charts in an Australian teaching hospital. They compared 312 documents (183 paper records vs. 129 electronic records) and internal audit data that included 8 paper audits with 3,834 paper-based records and 5 electronic audits with 2,958 electronic records. Completeness and legibility were analyzed and showed improved legibility of documentation (53·8% vs. 99·2%) ($p < 0·001$) and improved completeness of weight documentation (83·0% vs. 92·6%) ($p < 0·01$) in the EPR. The audit data showed that completeness improved in the documentation of mean height and weight (79·3% ± 3·8 vs. 86·0% ± 2·6) and malnutrition screening (57·5% ± 6·4 vs. 74·0% ± 8·1). There were no further statistical values or tests presented.

Montagna et al (2020) compared the trauma resuscitation documentation in 40 documents (20 paper records vs. 20 electronic records) in an hospital’s trauma center in Italy. They described a change in documentation style from a more narrative first-person documentation in the paper records to a list of events, including time and place in the EPR. They also found a lack of numerical values in 30% of the paper records and the documentation to be less accurate since many items were specified with an “about”, which did not show up in the electronic documentation. Volume of information was analyzed and showed a longer documentation of patient’s status at arrival in the EPR with 6·75 information in the paper records vs. 28·0 in the EPR. There were no further statistical values or tests presented.

Thoroddsen et al. (2011) compared 580 nursing care plans (299 paper records vs. 281 records 8 months after introduction of the EPR (195 electronic & 86 paper)) in an academic teaching hospital in Iceland. The use of the EPR was recommended but not mandatory, resulting in electronic and paper-based records in the second group. They analyzed the completeness and usage of standardized nursing terminology, measuring standardized nursing terminology with the North American Nursing Diagnosis Association (NANDA) classification for nursing diagnoses and Nursing Interventions Classification (NIC) for nursing interventions. Of the 299 paper records, 28% had a handwritten nursing care plan, 60% a preprinted nursing care plan and 12% did not have a nursing care plan at all. This changed to 70% computerized nursing care plans, 18% preprinted nursing care plans and 1% handwritten nursing care plans and still 11% that did not have a nursing care plan in the second group. They found a significantly higher number of nursing diagnoses in the EPR than in the preprinted nursing care plans of the second group (4·6 ± 2·3 vs. 5·9 ± 2·4) ($p < 0·001$), but not higher than in the remaining handwritten records of the second group. There was a significantly higher variety of diagnoses in the EPR (n = 91) than in the other two charting methods (n = 46 for preprinted and n = 22 for handwritten) ($p < 0·001$). Additionally, the number of nursing interventions was significantly higher in the EPR (11·7 ± 7·5) than in the other two charting methods (n = 7·1 ± 4·1 for preprinted and n = 1·8 ± 4·0 for handwritten) ($p < 0·001$). Of the tree documentation methods in the second group, the EPR had the highest completeness in documenting signs and symptoms (79% in the EPR vs. 63% in preprinted vs. 43% in handwritten).

Yadav et al. (2017) compared 500 physical examination notes (250 paper records vs. 250 electronic records) of patients treated with five ICD-9 codes in an US hospital. The diagnoses were permanent atrial fibrillation, aortic stenosis, intubation, lower limb amputation, and cerebrovascular accident (CVA) with hemiparesis. They analyzed

accuracy, inaccuracy, omission of and volume of information. All five ICD-9 codes come with certain conditions. Therefore, accuracy meant that the expected condition (e.g., missing of extremities) which is indispensably connected to the certain diagnose (e.g., lower limb amputation) is documented. Inaccuracy meant finding a healthy or the opposite of the expected condition documented, and omission of information meant that documentation of physical examination was missing. They found significantly higher rates of inaccuracy in the EPR (4·4% vs. 24·4%) ($p < 0{\cdot}001$) with more omitted information in the paper record (41·2% vs. 17·6%) ($p < 0{\cdot}001$). Moreover, the documentation in the EPR was significantly longer than in the paper record (15 words vs. 69 words) ($p < 0{\cdot}001$).

Zargaran et al. (2018) compared 20,848 documents (9,236 paper records vs. 11,612 electronic records) including admission notes, operation notes and discharge summaries in an academic teaching hospital in South Africa. They analyzed completeness and found all three record types to be significantly more complete in the EPR ($p < 0{\cdot}001$). The specific items in the admission notes were documentation of age (76% vs. 100%), sex (64% vs. 100%), injury date (64% vs. 100%), injury location (36% vs. 100%), injury mechanism (68% vs. 100%), and admission vital signs (68% vs. 100%). In the operative notes estimated blood loss (32% vs 100%), World Health Organization checklist completion (12% vs. 100%), postoperative disposition (4% vs. 100%), future plans (16% vs. 100%). And in discharge summaries length of stay (14% vs. 100%), intensive care unit admission (68% vs. 100%), complications (40% vs. 100%), missed injuries (20% vs. 100%). Although they stated statistical significance for all named items, they did not specify the p-value. Higher rates of completeness are assumed to be reached with mandatory entries in the EPR before the record can be closed.

Additional File 5: Mixed Methods Appraisal Tool

The Analyzation of Change in Documentation due to the Introduction of Electronic Patient Records in Hospitals - A Systematic Review

Authors
Florian Wurster[1]
Garret Fütterer[1]
Marina Beckmann[1]
Kerstin Dittmer[1]
Julia Jaschke[2]
Juliane Köberlein-Neu[2]
Mi-Ran Okumu[1]
Carsten Rusniok[1]
Holger Pfaff[1]
Ute Karbach[1]

Affiliations
[1] University of Cologne, Faculty of Human Sciences & Faculty of Medicine and University Hospital Cologne, Institute of Medical Sociology, Health Services Research, and Rehabilitation Science, Germany
[2] University of Wuppertal, Center for Health Economics and Health Services Research, Germany

Correspondence
Florian Wurster, M.Sc.
+49 221 478-97116
florian.wurster@uni-koeln.de
Eupener Str. 129
50933 Cologne, Germany

Additional File 5: Mixed Methods Appraisal Tool

5.1 Screening questions

First author	Year	S1. Are there clear research questions?	S2. Do the collected data allow to address the research questions?
Al Muallem	2017	Yes	Yes
Barritt	2010	Yes	Yes
Bell	2013	Yes	Yes
Boo	2012	Yes	Yes
Bruylands	2013	Yes	Yes
Choi	2014	Yes	Yes
Coffey	2015	Yes	Yes
Hampe	2017	Yes	Can't tell
Jamieson	2017	Yes	Yes
Jang	2013	Yes	Yes
Liu	2020	Yes	Yes
Lucas	2019	Yes	Yes
McCamley	2019	Yes	Yes
Montagna	2020	Yes	Yes
Thoroddsen	2011	Yes	Yes
Yadav	2017	Yes	Yes
Zargaran	2018	Yes	Yes

5.2 Randomized controlled trials

First author	Year	2.1. Is randomization appropriately performed?	2.2. Are the groups comparable at baseline?	2.3. Are there complete outcome data?	2.4. Are outcome assessors blinded to the intervention provided?	2.5 Did the participants adhere to the assigned intervention?	Sum Yes
Jamieson	2017	Yes	Yes	Yes	Yes	Yes	5

5.3 Non-Randomized Studies

First author	Year	3.1. Are the participants representative of the target population?	3.2. Are measurements appropriate regarding both the outcome and intervention (or exposure)?	3.3. Are there complete outcome data?	3.4. Are the confounders accounted for in the design and analysis?	3.5. During the study period, is the intervention administered (or exposure occurred) as intended?	Sum Yes
Al Muallem	2017	Yes	Yes	Yes	No	Yes	4
Barritt	2010	Can't tell	Yes	No	No	Yes	2
Bell	2013	Yes	Yes	Yes	No	Yes	4
Boo	2012	Yes	Can't tell	Yes	Yes	Yes	4
Choi	2014	Can't tell	Yes	Can't tell	No	Yes	2
Coffey	2015	Yes	Yes	Yes	Yes	Yes	5
Jang	2015	Yes	Yes	Yes	Yes	Yes	5
Liu	2013	Yes	Yes	Yes	Yes	Yes	5
Lucas	2019	Yes	Can't tell	No	No	No	1
McCamley	2019	Can't tell	No	No	Can't tell	Yes	1
Thoroddsen	2011	Yes	Yes	No	No	No	2
Yadav	2017	Yes	Yes	Yes	Yes	Yes	5
Zargaran	2018	Yes	Yes	Can't tell	Can't tell	Yes	3

5.4 Quantitative Descriptive Studies

First author	Year	4.1. Is the sampling strategy relevant to address the research question?	4.2. Is the sample representative of the target population?	4.3. Are the measurements appropriate?	4.4. Is the risk of nonresponse bias low?	4.5. Is the statistical analysis appropriate to answer the research question?	Sum Yes
Bruylands	2013	No	No	Yes	Yes	No	2
Hampe	2017	Can't tell	Can't tell	Yes	Yes	No	2

5.5 Mixed Method Studies

First author	Year	5.1. Is there an adequate rationale for using a mixed methods design to address the research question?	5.2. Are the different components of the study effectively integrated to answer the research question?	5.3. Are the outputs of the integration of qualitative and quantitative components adequately interpreted?	5.4. Are divergences and inconsistencies between quantitative and qualitative results adequately addressed?	5.5. Do the different components of the study adhere to the quality criteria of each tradition of the methods involved?	Sum Yes
Montagna	2020	Yes	Yes	No	No	No	2

Online Resource 6: A priori protocol

The Analyzation of Change in Documentation due to the Introduction of Electronic Patient Records in Hospitals - A Systematic Review

Authors
Florian Wurster[1]
Garret Fütterer[1]
Marina Beckmann[1]
Kerstin Dittmer[1]
Julia Jaschke[2]
Juliane Köberlein-Neu[2]
Mi-Ran Okumu[1]
Carsten Rusniok[1]
Holger Pfaff[1]
Ute Karbach[1]

Affiliations
[1] University of Cologne, Faculty of Human Sciences & Faculty of Medicine and University Hospital Cologne, Institute of Medical Sociology, Health Services Research, and Rehabilitation Science, Germany
[2] University of Wuppertal, Center for Health Economics and Health Services Research, Germany

Correspondence
Florian Wurster, M.Sc.
+49 221 478-97116
florian.wurster@uni-koeln.de
Eupener Str. 129
50933 Cologne, Germany

Online Resource 6: A priori protocol

Review Question

This systematic review follows the question of "Which approaches can analyze a change in documentation during the transition from paper-based to electronic documentation in hospitals?". It summarizes evidence from comparing paper-based and electronic patient records in hospital settings.

Searches

A sensitive search principle will be applied, in that databases will be searched with the means of finding all relevant articles published relating to the research question to be included in the review. Additionally, a manual search will be conducted to identify whether the following key words will be found by the search string: "QNOTE", "PDQI-9"/"PDQI-22", "Cat-ch-Ing".

Types of Included Studies

Studies included will be document analyses of paper and electronic records, randomized controlled studies, non-randomized studies, quantitative descriptive studies, qualitative studies or mixed-method studies.

Condition or Domain Being Studied

According to the PICOS-scheme, the components studied were

**P**roblem	**Implementation**
**I**ntervention	**electronic patient records**
**C**omparison	**paper-based** patient records
**O**utcome	change in **documentation** content or quality
**S**etting	**hospitals**

Inclusion criteria

Studies should meet the following criteria to be included: English or German language, published between 2010 and 2020, conducted in a hospital setting, focused on the transition between paper-based and electronic records, analysis of written documentation, primary data analysis, peer-reviewed.

Data Extraction

The following databases will be searched:

Database	Fields
CINAHL	Healthcare
MEDLINE, Pubmed & Pubmed Central via NCBI Search	Medicine
PDQ Evidence	Implementation research, healthcare systems, politics
Web of Science Core Collection	Science, technology, medicine

For the search process, synonyms for the search terms were identified utilizing a three step approach:

1. Brainstorming of terms through an oriented search process
2. Searching for synonyms using https://www.thesaurus.com

3. Singulars/plurals, abbreviations, different suffixes

Additionally, the term catalogues MeSH on Demand and Yale MeSH Analyzer of the PubMed database were searched for Medicinal Subject Headings (MeSH). Terms were checked by using the Advanced Search function of PubMed for indexation.
A PRISMA flow diagram will be made to visualize the screening process.

The following information will be extracted from studies:
- Study information: author, year, country
- Methods: study design
- Intervention: use case
- Outcomes and Results

Risk of Bias Assessment
Two reviewers will assess risk of bias independently, using the Mixed Methods Appraisal Tool (MMAT).
